# Ultra-Broadband Negative Photoconductivity in a Van der Waals Ferromagnetic Metal FePd_2_Te_2_

**DOI:** 10.3390/nano16191228

**Published:** 2026-09-29

**Authors:** Kang Zhang, Ruya Cong, Huixian Li, Feng Jiang, Tao Han, Xin Zhang, Jianzhi Gao, Yubing Tu, Suofu Wang, Mingsheng Long

**Affiliations:** 1 State Key Laboratory of Opto-Electronic Information Acquisition and Protection Technology, Institutes of Physical Science and Information Technology, Anhui University, Hefei 230601, China; 2 Key Laboratory of Opto-Electronic Information Acquisition and Manipulation of the Ministry of Education, Anhui University, Hefei 230601, China; 3 School of Physics and Information Technology, Shaanxi Normal University, Xi’an 710119, China; 4 School of Energy, Power and Safety, Anhui University of Applied Technology, Hefei 230011, China

**Keywords:** negative photoconductivity, photobolometric effect, FePd_2_Te_2_, photodetectors

## Abstract

Negative photoconductivity (NPC) in low-dimensional materials has attracted considerable interest for its potential in novel optoelectronic devices. Here, we report an ultra-broadband NPC response in FePd_2_Te_2_, a van der Waals ferromagnet, spanning from visible to long-wavelength infrared at room temperature. The device achieves a responsivity of ~4.9 A/W and external quantum efficiency of ~240% at 2500 nm, with a noise-equivalent power (*NEP*) below 3.0 × 10^−15^ W/Hz^1/2^ and a specific detectivity (D*) exceeding 3.6 × 10^11^ cmHz^1/2^/W across the infrared range. Systematic measurements identify the photobolometric effect as the underlying mechanism, wherein wavelength-dependent absorption depths play a key role in shaping the photoresponse characteristics. These results establish FePd_2_Te_2_ as a promising platform for uncooled broadband infrared detection and highlight the potential of magnetic van der Waals materials for future optoelectronics.

## 1. Introduction

Negative photoconductivity (NPC) is a phenomenon in which the electrical signal intensity decreases with increasing light intensity, as opposed to positive photoconductivity (PPC). Under normal circumstances, illumination enhances a material’s conductivity, and the resulting photocurrent increases proportionally with light intensity. Recent studies, however, have revealed that certain low-dimensional materials exhibit an intriguing opposite behavior known as NPC, where the photogenerated current falls below the reference dark current [1,2]. For example, in uncooled InAs nanowire metal–semiconductor–metal photodetectors, the naturally formed InO_x_ shell layer acts as a photo-gating layer that traps photogenerated carriers and depletes the nanowire channel [3]. Under 405 nm illumination, the device achieves a dark-to-photocurrent ratio of ~130, an NPC gain as high as 10^5^ at low bias, and a fast rise time of 0.005 s, demonstrating its potential for visible/near-infrared optical communication applications. Similarly, monolayer MoS_2_ has been reported to exhibit trion-induced NPC, with conductivity dropping to ~30% of its equilibrium value [4]. MoS_2_-based heterostructures further enrich the NPC landscape: MoS_2_/Ge shows switchable PPC/NPC under visible versus infrared light [5]; MoS_2_/Ti_3_C_2_T_x_ exhibits a bipolar photoresponse with NPC at 700–800 nm [6]; and ReS_2_/hBN/MoS_2_ floating-gate structures achieve NPC through interfacial charge transfer [7]. Graphene also displays NPC through the photobolometric effect with ultrahigh bandwidth [8]. Heterostructures further extend the tunability: graphene/SnS_2_ leverage deep-level traps for wavelength-dependent NPC [9], while Bi_2_O_2_Se/graphene achieves polarity-switchable PPC/NPC through the competition between water/oxygen desorption and thermal electron injection [10]. In low-dimensional systems, photocurrent spectroscopy has long been used to determine band gaps and excitonic transitions, as demonstrated in quantum-well heterostructures [11]. These unique characteristics have attracted considerable attention owing to their potential applications in photoelectric detection [12,13,14,15], logic devices [16,17], neuromorphic computing [18,19], and low-power nonvolatile memory [20,21]. Taken together, this material diversity underscores NPC as a versatile and tunable phenomenon across low-dimensional systems, alongside other anomalous transport phenomena such as negative differential resistance [22], establishing a rich platform for designing next-generation optoelectronic devices with reconfigurable, multifunctional capabilities.

FePd_2_Te_2_ has recently been reported as a van der Waals ferromagnetic material, featuring a layered quasi-2D crystal structure with unique quasi-one-dimensional Fe zigzag chains and a Curie temperature of ~183 K [23]. Structurally and chemically, FePd_2_Te_2_ can be regarded as a magnetic derivative of PdTe_2_, a well-known type-II Dirac semimetal that has been extensively employed in photodetectors. PdTe_2_-based devices have demonstrated detection ranging from deep ultraviolet through visible to mid-infrared [24,25,26]. These detectors consistently achieve exceptional performance: specific detectivities reaching 10^13^ Jones [27], ultrafast response times down to microseconds [28], and room-temperature imaging capability [26,29]. These attributes make PdTe_2_ one of the most versatile 2D material platforms for broadband optoelectronic applications. Similarly, Fe_3_GeTe_2_, another extensively studied van der Waals ferromagnet, has also emerged as a key material in optoelectronics and spintronics. It features a rich topological electronic structure, gate-tunable room-temperature ferromagnetism, anomalous quantum Hall effect, and skyrmion physics [30,31,32]. Fe_3_GeTe_2_ exhibits tunable PPC/NPC that can be switched by light power, bias, and temperature [33]. Its heterostructures have been explored for terahertz (THz) spin current generation [34]. For example, Fe_3_GeTe_2_/O-Fe_3_GeTe_2_ heterostructures have achieved broadband NPC from ultraviolet to THz (responsivity 2.572 A/W at 0.1 THz, *NEP* 26 pW Hz^1/2^) [35]. These results establish it as a platform where magnetism and photonics converge. Indeed, FePd_2_Te_2_ also exhibits prominent THz photoelectric emission when integrated with Pt, indicating efficient light-driven spin-to-charge conversion [36]. Furthermore, its robust air stability and mechanical exfoliability down to few-layer thicknesses further position it as a uniquely promising candidate for exploring photoelectric phenomena.

Here, we report an ultra-broadband NPC response in FePd_2_Te_2_-based photodetectors operating from visible (637 nm) to long-wavelength infrared (LWIR, 10.6 μm) at room temperature. The device exhibits a symmetric photoresponse under opposite bias polarities, with responsivities reaching ~4.9 A/W and external quantum efficiencies up to 240% in the mid-infrared range. Through systematic wavelength- and power-dependent measurements, we identify the photobolometric effect as the dominant mechanism underlying this broadband NPC, which is further corroborated by the metallic temperature dependence of resistivity in FePd_2_Te_2_. While broadband NPC has been reported in Fe_3_GeTe_2_-based native heterostructures through an interface-assisted dual-response mechanism [35], and PdTe_2_ has demonstrated excellent infrared photodetection via photovoltaic effects [24,25,26], FePd_2_Te_2_ may offer a distinct platform: a van der Waals layered metal whose broadband NPC is consistent with a photobolometric mechanism, yielding bias-symmetric, room-temperature operation with low *NEP* and high D* across the infrared.

## 2. Materials and Methods

FePd_2_Te_2_ single crystals were grown by solid-state reaction. Iron powder, tellurium powder, and palladium lumps were weighed in a molar ratio of 1:2:2, placed in an alumina crucible, and sealed in a quartz tube under vacuum. The entire quartz tube was placed in a box furnace, heated from room temperature to 850 °C over 5 h, and held at that temperature for 2 days. It was then cooled to 600 °C at a rate of 2 °C/h, annealed at this temperature for 2 days, and then furnace-cooled to room temperature. Single-crystal X-ray diffraction (XRD) patterns were collected by Rigaku SmartLab (Rigaku Corp., Tokyo, Japan) with Cu-Kα1 radiation at room temperature. The thickness of the FePd_2_Te_2_ nanoflake was characterized using an atomic force microscope (AFM, Oxford, Santa Barbara, CA, USA). Energy-dispersive X-ray spectroscopy (EDS) elemental analysis was carried out using a Zeiss Crossbeam 550 L system (Carl Zeiss Microscopy GmbH, Oberkochen, Germany). Magnetization and electrical transport measurements on bulk samples were carried out in Quantum Design MPMS3 and PPMS, respectively.

The prefabricated bottom Au electrodes were fabricated using standard electron-beam lithography and thermal evaporation deposition of Cr/Au (5/30 nm) on a heavily doped Si substrate (0.5 mm, orientation: 〈100〉, resistivity 0.01–0.05 Ω cm, Hefei Kejing Material Technology Co., Ltd., Hefei, China) covered by 300 nm SiO_2_. Thermal evaporation was performed using a VZZ-400 system (VNANO Co., Ltd., Beijing, China). Electrical properties and photoresponse tests were conducted using a dual-channel digital source meter (Keithley 2636B, Cleveland, OH, USA). The 637 nm excitation laser was focused onto the device through a 20× objective lens. For investigating the detector performance in the mid-wavelength infrared (MWIR) region, a wavelength-tunable multi-channel MWIR laser (2.5–4.2 μm) manufactured by Nanjing Nannzhi Helight Technology Co., Ltd. (Nanjing, China) was employed, with a laser beam diameter of ∼4.4 mm. The LWIR (10.6 μm) performance was studied using a CO_2_ laser source. All measurements were performed in ambient air at room temperature.

## 3. Results and Discussion

**Figure 1:**
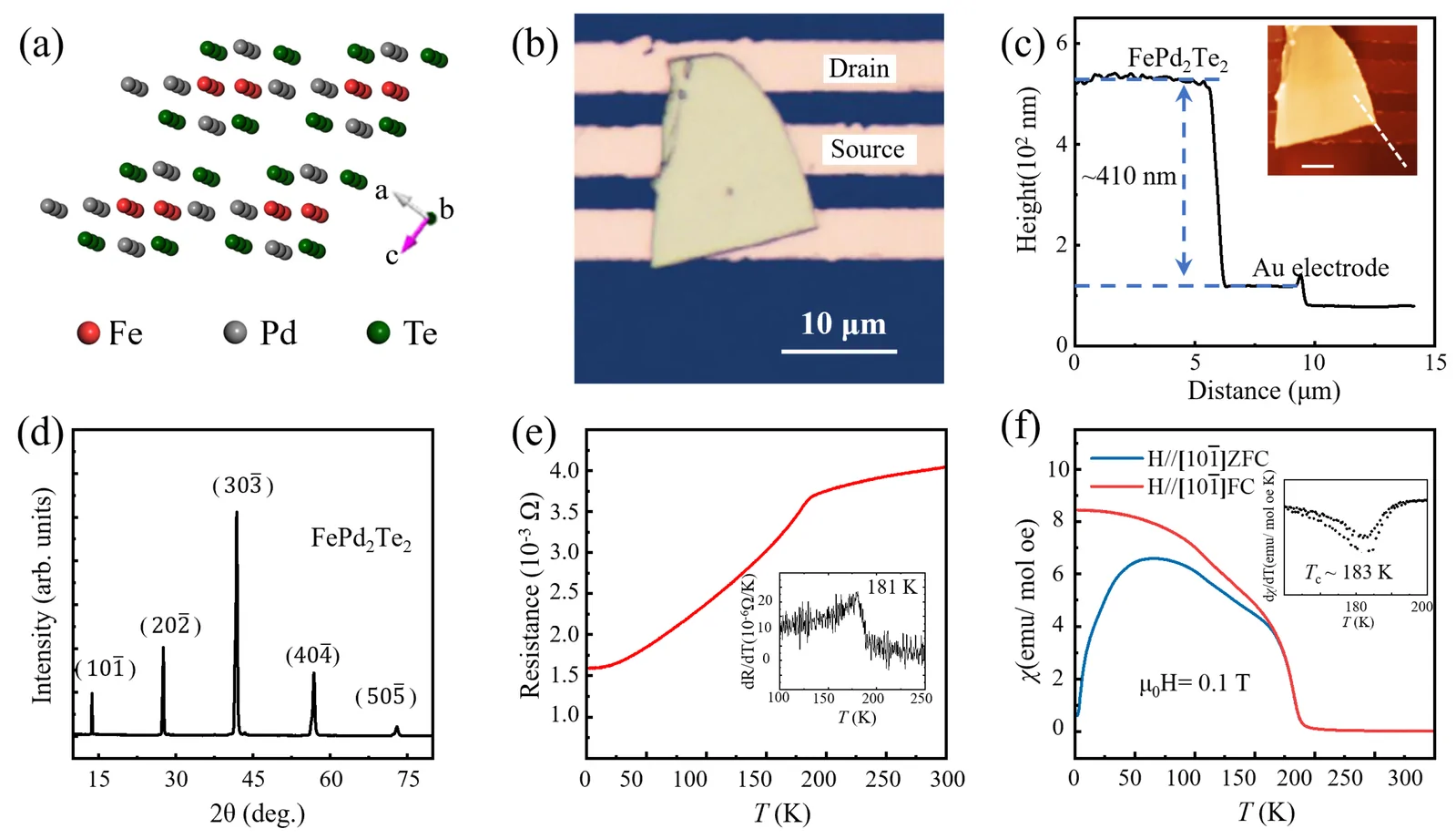
Characterizations of the FePd_2_Te_2_ single crystal and the device configuration. (**a**) Crystallographic structure of FePd_2_Te_2_. (**b**) Optical image of the FePd_2_Te_2_ nanoflake on the bottom electrode. (**c**) The step-height profiles of the FePd_2_Te_2_ flake. The inset is the atomic force microscope image of the FePd_2_Te_2_ device (the scale bar is 5 μm). (**d**) X-ray diffraction pattern of the FePd_2_Te_2_ crystal. (**e**) Temperature-dependent resistivity of the FePd_2_Te_2_ single crystal. The inset shows the derivative of the resistivity curve. (**f**) Temperature-dependent zero-field-cooled (ZFC) and field-cooled (FC) magnetic susceptibility of the FePd_2_Te_2_ single crystal at μ_0_H = 0.1 T. The inset shows the derivative of the magnetic susceptibility.

The vdW ferromagnet FePd_2_Te_2_ crystallizes in a monoclinic structure (space group *P*2_1_/*m*), as shown in Figure 1a. This structure features an ABC-type stacking sequence of Te–Pd/Fe–Pd/Te–Pd trilayers that enables facile exfoliation. The X-ray diffraction pattern of the FePd_2_Te_2_ flake is presented in Figure 1d. It displays sharp ($n0\bar{n}$) peaks with narrow full width at half-maximum, confirming that the flake grows precisely along the ($10\bar{1}$) direction. The temperature-dependent resistivity, shown in Figure 1e, exhibits metallic behavior over the entire measured range, with a distinct anomaly near the magnetic transition (~181 K, inset of Figure 1e). This transition is further corroborated by magnetization measurements (Figure 1f), which reveal a paramagnetic-to-ferromagnetic transition at ~183 K. When the magnetic field is applied perpendicular to the cleavage plane, the magnetization exhibits a sharp upturn at T_c_ and a pronounced bifurcation between zero-field-cooled and field-cooled curves. This bifurcation is commonly observed in layered ferromagnets like Fe_3_GeTe_2_, where it has been attributed to domain-wall pinning and depinning processes [37]. In addition, EDS characterization of the FePd_2_Te_2_ nanoflake is presented in Figure S1. These results are in good agreement with previous reports and confirm the high quality of our single crystals [23]. The unique properties of FePd_2_Te_2_ suggest this material is a promising platform for exploring optoelectronic phenomena.

Using the dry transfer technique, the FePd_2_Te_2_ nanoflake was placed on the pre-deposited bottom Au electrode. Figure 1b presents the optical photograph of the nanoflake. The surface morphology and thickness were further characterized by atomic force microscopy (AFM). As shown in the AFM image (inset of Figure 1c), the flake exhibits a smooth surface. The step-height profile across the edge yields a thickness of ~410 nm, as shown in Figure 1c. Motivated by the metallic transport behavior and high crystal quality, we systematically investigated the photoelectric response of the device.

**Figure 2:**
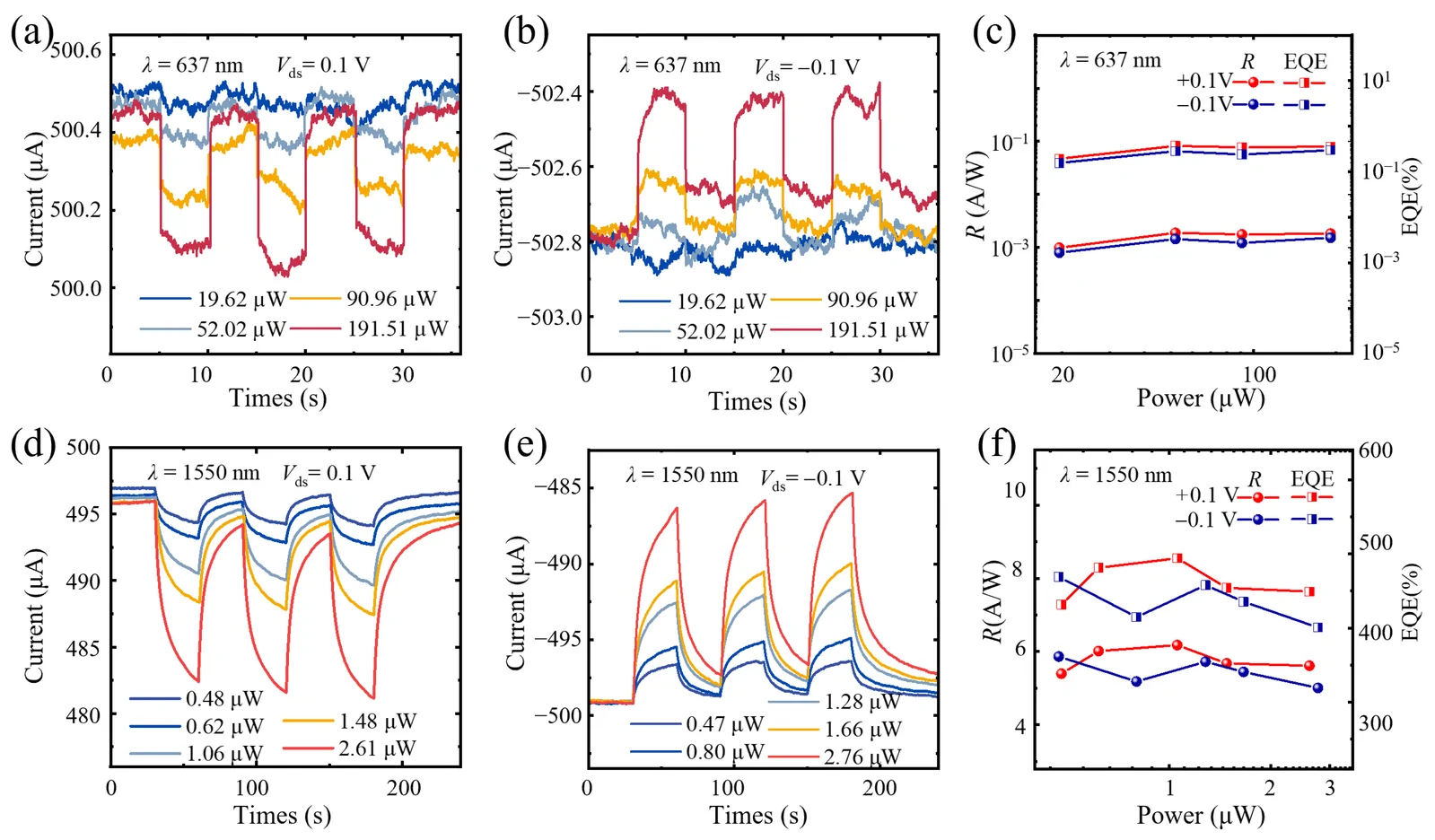
Negative photoresponse of the FePd_2_Te_2_ device. (**a**,**b**) Temporal photoresponse of the FePd_2_Te_2_ device under 637 nm illumination at +0.1 V and −0.1 V bias. (**c**) Calculated *R* and EQE as a function of incident light power under 637 nm illumination. (**d**,**e**) Temporal photoresponse of the FePd_2_Te_2_ device under 1550 nm illumination at +0.1 V and −0.1 V bias. (**f**) Calculated *R* and EQE as a function of incident light power under 1550 nm illumination.

We first measured the temporal photoresponse under 637 nm and 1550 nm laser excitation. Under visible 637 nm illumination with ±0.1 V bias, the device displays a clear NPC effect. At low incident power, the photogenerated signal is relatively small, making the absolute current change modest compared with the large dark current. However, as the incident power rises, a pronounced current decrease at +0.1 V and a current increase at −0.1 V are resolved (Figure 2a,b). Moreover, this photocurrent variation upon laser switching is highly reproducible. This periodic power-dependent negative photoresponse confirms a light-induced reduction in conductance—a hallmark of NPC. Data from another independently fabricated device are provided in Figure S3, showing consistent NPC behavior. To quantify the photo-to-current conversion efficiency, we calculated the photoresponsivity (*R* = *I*_p_/*P*) and external quantum efficiency (EQE = *hcR*/*eλ*), where *I*_p_ = *I*_Light_ − *I*_Dark_ is the photocurrent, *P* is the effective incident power corrected by the device-to-beam area ratio, *h* is Planck’s constant, *c* is the speed of light, *λ* is the wavelength, and *e* is the electron charge. The power-dependent *R* and EQE under 637 nm illumination are shown in Figure 2c. Both *R* and EQE exhibit weak dependence on the incident power.

The time-resolved photoresponses at ±0.1 V under 1550 nm were also measured, as displayed in Figure 2d and e, respectively. Upon 1550 nm irradiation, the current substantially decreased at positive bias and increased at negative bias, recovering when the light was switched off. This behavior—identical to that observed under 637 nm excitation—confirms that the NPC response persists under both bias polarities, ruling out contributions from asymmetric electrode contacts or the photothermoelectric effect. However, the photoresponse time is longer than that under visible light, which we will discuss later. The power-dependent *R* and EQE under 1550 nm illumination were also calculated and are plotted in Figure 2f. As the wavelength increased from 637 nm to 1550 nm, both *R* and EQE remained weakly dependent on the incident power, similar to the visible case. Meanwhile, *R* and EQE are distinctly higher compared with those at 637 nm, indicating a stronger NPC response at longer wavelengths in terms of both responsivity and relative current change. These results prompt further investigation of the NPC response at other wavelengths.

**Figure 3:**
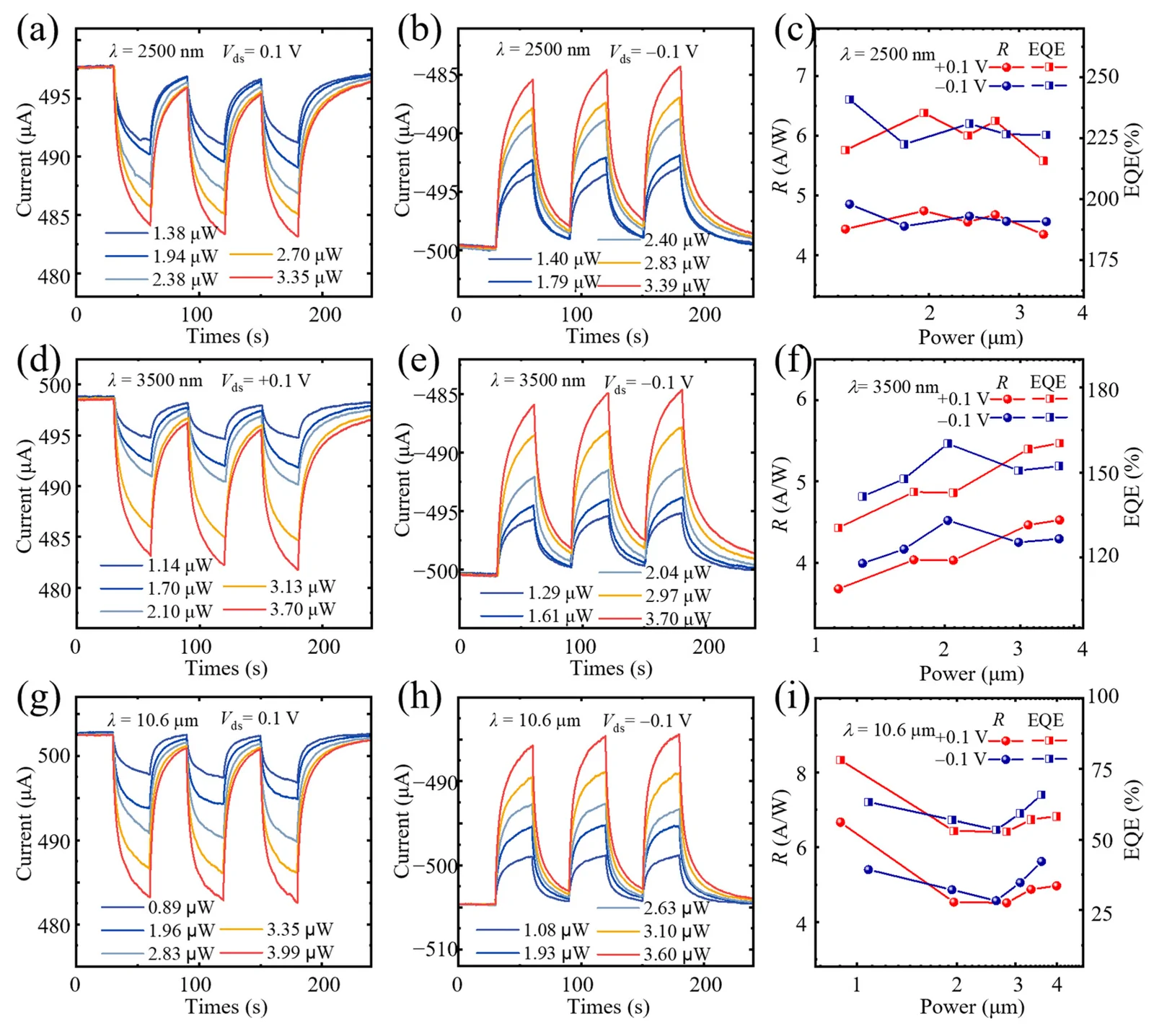
Optoelectric properties of the FePd_2_Te_2_ device under 2500 nm, 3500 nm, and 10.6 μm illumination. (**a**,**b**) Temporal photoresponse of the FePd_2_Te_2_ device under 2500 nm illumination at +0.1 V and −0.1 V bias. (**c**) Calculated *R* and EQE as a function of incident light powers under 2500 nm illumination. (**d**,**e**) Temporal photoresponse of the FePd_2_Te_2_ device under 3500 nm illumination at +0.1 V and −0.1 V bias. (**f**) Calculated *R* and EQE as a function of incident light powers under 3500 nm illumination. (**g**,**h**) Temporal photoresponse of the FePd_2_Te_2_ device under 10.6 μm illumination at +0.1 V and −0.1 V bias. (**i**) Calculated *R* and EQE as a function of incident light power under 10.6 μm illumination.

We next investigated the device performance in the mid-wave to long-wave infrared spectral range. The temporal photoresponse under 2500 nm illumination at ±0.1 V is shown in Figure 3a,b. Upon laser illumination, the current decreased at +0.1 V and increased at −0.1 V, consistent with the NPC behavior observed at shorter wavelengths. For 2500 nm, a photocurrent of −13.6 μA (14.4 μA) was obtained under 3.35 μW (3.39 μW) light power at +0.1 V (−0.1 V). The comparable magnitudes at opposite biases again confirm that the NPC response is nearly symmetric under the two polarities. The corresponding power-dependent *R* and EQE at ±0.1 V bias under a 2500 nm laser are shown in Figure 3c. Both *R* and EQE exhibit weak power dependence, analogous to the trend observed under 1550 nm. Under 2500 nm, an *R* of 4.9 (4.8) A/W and a corresponding EQE of 240.0% (235.0%) were achieved at +0.1 V (−0.1 V). The temporal photoresponse of the device at ±0.1 V under various illumination powers at 3500 nm is shown in Figure 3d,e. The current change induced by NPC is comparable to that at 2500 nm, and the photocurrent scales with light power at both bias polarities. The power-dependent *R* and EQE are plotted in Figure 3f. In contrast to the behavior at 2500 nm, the extracted *R* and EQE at 3500 nm increase slightly but monotonically with rising light power. An *R* value of 4.5 A/W with an EQE of 160.0% was achieved under a light power of 3.7 μW.

Remarkably, as the wavelength extends to 10.6 μm, the periodic current decrease and increase upon light switching confirm that the NPC effect remains strong in the LWIR range (Figure 3g,h). The *R* and EQE show a nonlinear dependence on the incident power, which differs markedly from the trends observed at other wavelengths. At the lowest excitation power of ~1 μW, both *R* and EQE attain their maxima. These values then decline as the power approaches ~2.5 μW, followed by a gradual recovery upon further increasing the power.

So far, we have demonstrated that the FePd_2_Te_2_-based photodetector exhibits an ultra-broadband photoresponse ranging from visible to LWIR. However, the mechanism underlying this broadband NPC remains to be elucidated. Several NPC mechanisms have been proposed, including the photobolometric effect, carrier trapping, the trionic effect, adsorption–desorption of surface molecules, and localized surface plasmon resonances [1,2,3,4,10,12,38,39,40]. The carrier trapping mechanism involves defect states that trap photogenerated carriers, and the resulting localized trapped charges effectively modulate the channel conductance [3,39]. This mechanism is, however, inconsistent with the metallic character of FePd_2_Te_2_, where the high carrier concentration provides strong electrostatic screening, rendering trapped charges ineffective. The trionic effect is also ruled out by the ultra-broadband response from 637 nm to 10.6 μm, as trion formation requires resonant excitation and cannot account for a broadband response [4]. Moreover, FePd_2_Te_2_ exhibits a non-zero density of states at the Fermi level and lacks excitonic states, which precludes the possibility of trion formation. Likewise, the adsorption–desorption of surface molecules and localized surface plasmon resonances each impose specific requirements on the device structure: the former requires a particular surface chemistry [10,40], while the latter relies on the presence of metal nanoparticles [41]. Neither condition is satisfied in our experimental configuration. Instead, we propose that the photobolometric effect is the most likely mechanism underlying the ultra-broadband NPC in FePd_2_Te_2_-based photodetectors. This assignment is strongly supported by the metallic temperature dependence of resistivity shown in Figure 1e and the current-voltage curves of the device in Figure S2. The former provides the necessary condition for a positive resistance change upon light-induced heating.

**Figure 4:**
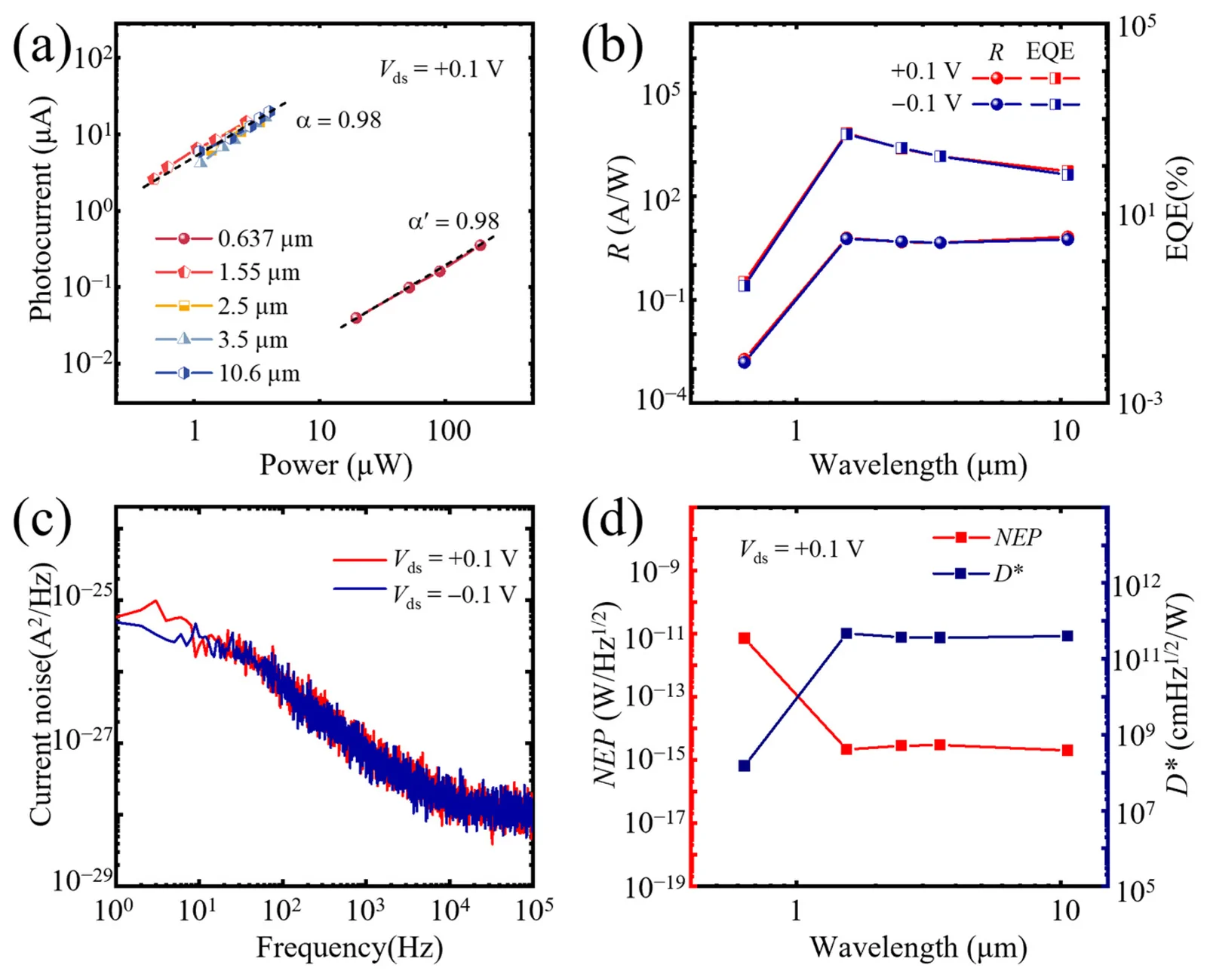
Broadband photodetection performance of the FePd_2_Te_2_ device. (**a**) Extracted photocurrents as a function of incident light power for different laser wavelengths at +0.1 V bias. (**b**) Calculated *R* and EQE as a function of laser wavelength. (**c**) Noise power spectral density versus frequency for the FePd_2_Te_2_ device. (**d**) Calculated *NEP* and D* as a function of laser wavelength.

Within the photobolometric effect framework, when light is incident on the material, the lattice temperature increases via electron-phonon coupling, and the resulting resistance increase directly produces the NPC [8,12,38]. This thermally induced resistance change is independent of the bias polarity, so the photocurrent under opposite bias polarities exhibits the reverse changes, as observed experimentally. Furthermore, the NPC arising from the photobolometric effect is intrinsically broadband—as long as the material absorbs light, heat is generated, and the photocurrent depends primarily on the absorbed optical power rather than the photon energy. To verify this, we measured the photocurrent as a function of incident light power at various wavelengths, as shown in Figure 4a (because the NPC response is nearly symmetric under the two bias polarities, only the +0.1 V case is displayed for clarity). Indeed, at the same incident power, the photocurrents are comparable across all wavelengths except the visible range. Consistently, a power-law behavior, *I*_p_∝*P*^α^, is observed for all wavelengths, with the power exponent α close to 1, indicating that the photocurrent is approximately proportional to the incident light power (*I*_p_∝*P*). This implies that across the infrared range (1500 nm–10.6 μm), the responsivity *R* (the ratio of photocurrent to incident power) is weakly dependent on wavelength, as shown in Figure 4b.

Notably, the photocurrent at 637 nm is significantly lower than that at longer wavelengths. This is because the absorption depth at 637 nm is very shallow [36], confining the photogenerated heat to a thin surface layer where it is rapidly dissipated through the substrate. Consequently, the temperature rise is limited, resulting in a small photocurrent and a fast response. For longer wavelengths, in contrast, the volumetric heating is more uniform, and heat dissipation is slower due to the longer thermal diffusion path. As a result, the temperature rise is larger, yielding a more pronounced photoresponse along with a longer response time. These behaviors are consistent with the temporal photoresponse shown in Figure 2 and Figure 3. Consistently, the weaker NPC at 637 nm also accounts for the low responsivity *R* and EQE at this wavelength in Figure 4b.

Another notable feature is that the EQE reaches its maximum at 1550 nm and then decreases with increasing wavelength. Although the photobolometric effect is intrinsically wavelength-independent and the responsivity *R* exhibits little variation across the infrared range, the photon energy *hν* decreases at longer wavelengths. Since EQE is proportional to the product of the photon energy and the responsivity, the EQE decreases monotonically with increasing wavelength in the long-wavelength regime, despite the deeper light penetration [36]. This wavelength-dependent absorption picture also explains the distinct power-dependent behaviors of *R* and EQE across different wavelengths (Figure 3). At shorter wavelengths, shallow absorption and rapid heat dissipation keep the temperature rise approximately linear with the incident power, so *R* remains nearly constant. At 3500 nm, deeper penetration may lead to a longer thermal diffusion path and a higher thermal resistance, making the temperature rise slightly superlinear and thus causing *R* and EQE to rise monotonically. At 10.6 μm, the effective thermal resistance is expected to be relatively high, so that the temperature rise may become increasingly nonlinear with power, and several possible thermal processes—such as interface conductance and radiative contributions—may compete, leading to the observed non-monotonic response. A full quantitative description would require dedicated thermal modeling.

To evaluate the sensitivity of the FePd_2_Te_2_ device, we measured the current noise power spectrum at ±0.1 V bias, as shown in Figure 4c. The 1/f noise dominates the current noise up to ~7 kHz, above which the noise power spectral density becomes frequency-independent, reaching a level of ~1 × 10^−28^ A^2^ Hz^−1^. The noise equivalent power and specific detectivity were then calculated to assess the capability of the device to extract weak optical signals from background noise. *NEP* = <*i*_n_^2^>^1/2^/*R* and D* = (*S*Δ*f*)^1/2^/*NEP*, where *S* is the effective area of the device, and Δ*f* is the measurement bandwidth, ${\langle {i_{n}}^{2}\rangle }^{1/2}=1/B\int_{0}^{B}S(f)\mathrm{df}$. As shown in Figure 4d, in the infrared range, the *NEP* remains below 3.0 × 10^−15^ W/Hz^1/2^ and D* exceeds 3.6 × 10^11^ cmHz^1/2^/W. This high level of performance is remarkably consistent from the near-infrared to the LWIR, demonstrating the high detection sensitivity of the FePd_2_Te_2_ device. The performance of FePd_2_Te_2_ is compared with that of other broadband photodetectors in Table S1.

## 4. Conclusions

We have demonstrated room-temperature ultra-broadband NPC in FePd_2_Te_2_-based photodetectors spanning from visible to LWIR. The device achieves a responsivity of ~4.9 A/W and an external quantum efficiency of ~240% at 2.5 μm, with a nearly symmetric photoresponse under opposite bias polarities. The low *NEP* and high D* demonstrate its high detection sensitivity across the infrared range. A combination of wavelength- and power-dependent measurements, along with the metallic behavior of FePd_2_Te_2_, identifies the photobolometric origin of the observed NPC. Notably, the wavelength-dependent absorption depth plays a critical role in determining the photoresponse magnitude, response time, and power-dependent behavior of *R* and EQE. These results establish FePd_2_Te_2_ as a promising platform for broadband, room-temperature infrared detection. Despite the relatively slow thermal response and possible substrate contribution, future thickness engineering and suspended device designs could further resolve the intrinsic photoresponse and expand the potential of magnetic van der Waals materials for optoelectronics.

## Data Availability

The raw data supporting the conclusions of this article will be made available by the authors on request.

## Supplementary Materials

The following supporting information can be downloaded at: https://www.mdpi.com/article/10.3390/nano16191228/s1, Figure S1. EDS characterization of the FePd_2_Te_2_ nanoflake. (a) EDS spectrum of the nanoflake. (b–d) Elemental mapping of the measured FePd_2_Te_2_ nanoflake, Fe (b), Te (c), and Pd (d). Figure S2. Current-voltage curves of the FePd_2_Te_2_ device under incident light of various wavelengths. (a) 637 nm, (b) 1550 nm, (c) 2550 nm, (d) 3500 nm, (e) 10.6 μm, and (f) magnified view of the dashed box region in (e). Figure S3. Optoelectronic response of another FePd_2_Te_2_ photodetector under 1550, 2500, and 3500 nm illumination. (a–e) Time-resolved photoresponse of the device under various incident powers at 637 nm, 1550 nm, 2500 nm, 3500 nm, and 10.6 μm, respectively. (f) Optical image of the FePd_2_Te_2_ nanoflake. Table S1. Comparison of the performance between FePd_2_Te_2_ and other broadband photodetectors. References [35,42] are cited in the Supplementary Materials.
