# Supplementary material for: Ultra-Broadband Negative Photoconductivity in a Van der Waals Ferromagnetic Metal FePd2Te2
Source: Nanomaterials (Basel). 2026 Sep 29;16(19):1228. doi: 10.3390/nano16191228 (PMC13666988; doi:10.3390/nano16191228)
Supplement: Supplementary file 1 [file nanomaterials-16-01228-s001.zip › nanomaterials-4588386-supplementary.pdf]

Ultra-Broadband Negative Photoconductivity in a Van der Waals Ferromagnetic Metal $FePd_2Te_2$

Kang Zhang [1], Ruya Cong [1], Huixian Li [1], Feng Jiang [1], Tao Han [1,2], Xin Zhang [3], Jianzhi Gao [3], Yubing Tu [1,2,*], Suofu Wang [4,*] and Mingsheng Long [1,2,*]

1 State Key Laboratory of Opto-Electronic Information Acquisition and Protection Technology, Institutes of Physical Science and Information Technology, Anhui University, Hefei 230601, China

2 Key Laboratory of Opto-Electronic Information Acquisition and Manipulation of the Ministry of Education, Anhui University, Hefei 230601, China

3 School of Physics and Information Technology, Shaanxi Normal University, Xi'an 710119, China

4 School of Energy, Power and Safety, Anhui University of Applied Technology, Hefei 230011, China

* Correspondence: tuyubing@ahu.edu.cn (Y.T.); wangsuofu@uta.edu.cn (S.W.); longms@ahu.edu.cn (M.L.)

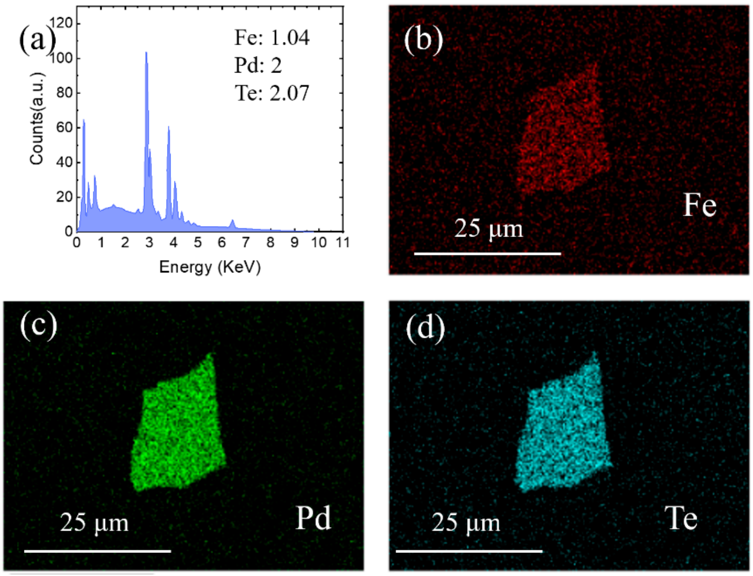

Figure S1. EDS characterization of the $FePd_2Te_2$ nanoflake. (a) EDS spectrum of the nanoflake. (b-d) Elemental mapping of the measured FePd2Te2 nanoflake, Fe(b), Te(c), and Pd(d).

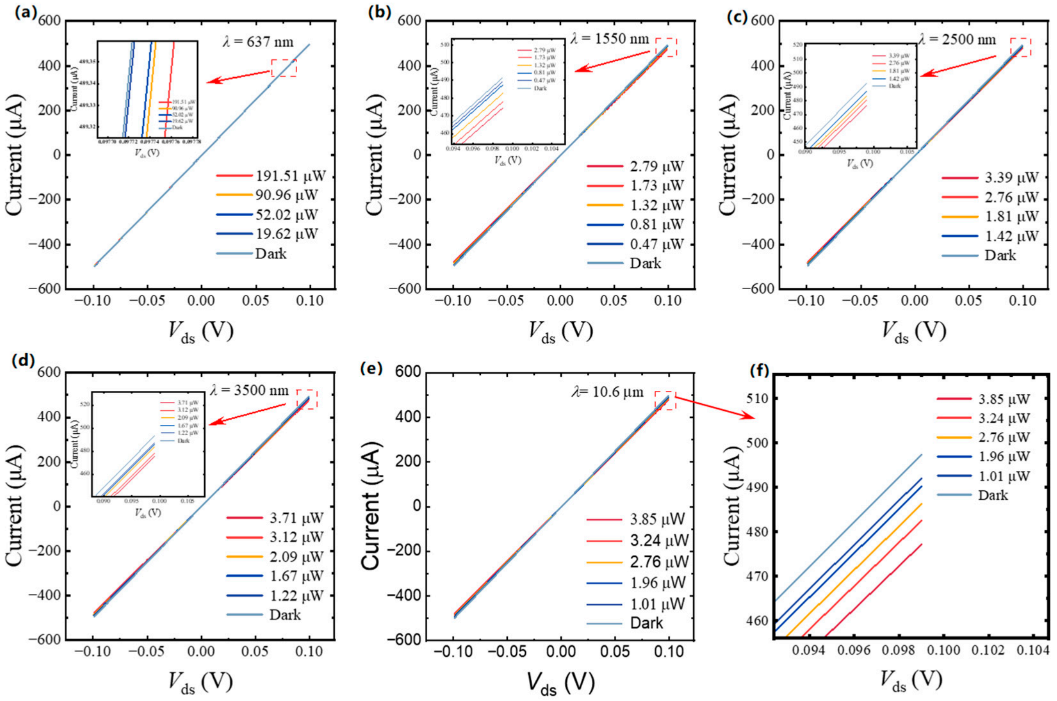

Figure S2. Current-voltage curves of the $FePd_2Te_2$ device under incident light of various wavelengths. (a) 637 nm, (b) 1550 nm, (c) 2550 nm, (d) 3500 nm, (e) 10.6 µm, and (f) magnified view of the dashed box region in (e).

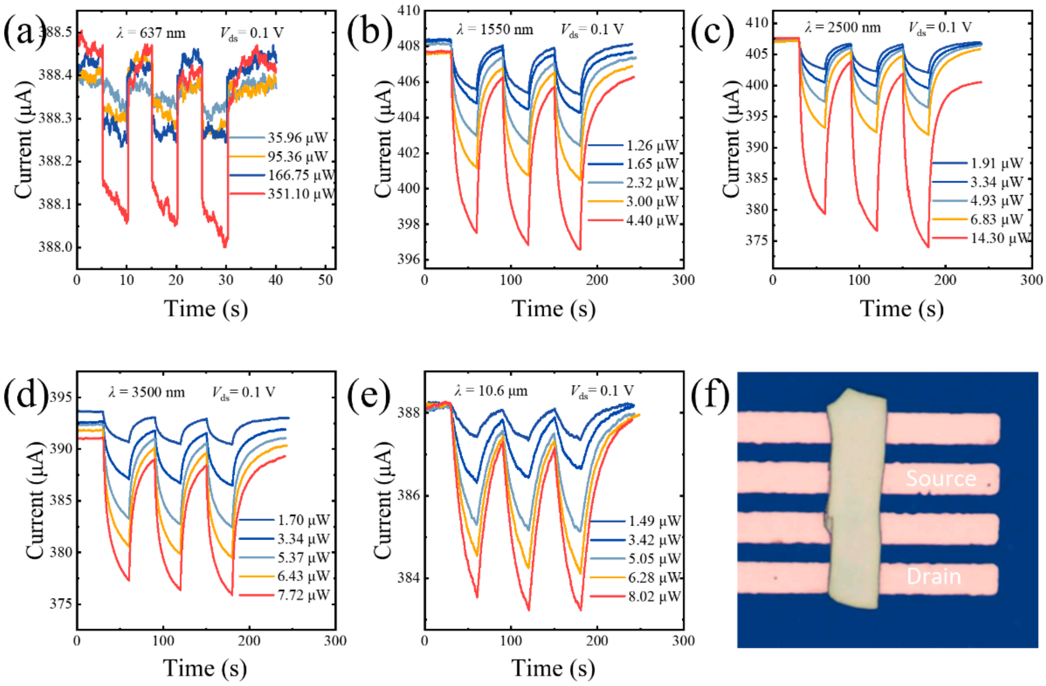

Figure S3. Optoelectronic response of another $FePd_2Te_2$ photodetector under 1550, 2500, and 3500 nm illumination. (a)-(e) Time-resolved photoresponse of the device under various incident powers at 637nm, 1550 nm, 2500 nm, 3500 nm, and 10.6μm, respectively. (f) Optical image of the $FePd_2Te_2$ nanoflake.

Table S1. Comparison of the performance between FePd2Te2 and other broadband photodetectors.

Materials	Bias(V)	Detection range	Response wavelength	D* ($cm\cdot Hz^{1/2}\cdot W^{-1}$)	NEP ($pW\ Hz^{-1/2}$)	Response time	Thickness	Temperature	Mechanism	Ref
Fe_3GeTe_2 /O-Fe_3GeTe_2	±0.1	35 nm-3000 μm	10.6 μm	7.2×10^6	24637	422 ms	134nm O-FGT	RT	Interface-assisted dual-response	35
$PtTe_2$	0.1	420 nm-10.6 μm	10.7 μm	2.6×10^7	--	--	~3 nm	RT	photoconductive effect and Photobolometric	42
$FePd_2Te_2$	±0.1	637 nm-10.6 μm	10.6 μm	3.99×10^{11}	1994	--	~410 nm	RT	photobolometric	This work
